# AGE-RELATED CHANGES IN BODY WEIGHT AS DETERMINANTS OF EXTREME LONGEVITY

**DOI:** 10.1093/geroni/igae098.2618

**Published:** 2024-12-31

**Authors:** Svetlana Ukraintseva, Hongzhe Duan, Rachel Holmes, Konstantin Arbeev, Anatoliy Yashin

**Affiliations:** Duke University, Durham, North Carolina, United States; Duke University, Durham, North Carolina, United States; Duke University, Durham, North Carolina, United States; Duke University, Durham, North Carolina, United States; Duke University, Durham, North Carolina, United States

## Abstract

Age-related changes in body weight may mirror fundamental processes of aging and ontogeny that contribute to determining human longevity limits. We estimated the mean age trajectories of weight, along with their key characteristics, such as the age at which maximum weight values (AgeMax) are reached, and the slope of weight decline (Slope) thereafter, and tested whether they can predict extreme longevity in the Framingham Heart Study participants. We compared weight trajectories over a period of approximately 50 years between those who survived beyond age 95 (longevity group) and those who died before age 85 (conventional lifespan). We also estimated survival trajectories after age 80 for individuals who had different AgeMax and Slope. We found that individuals who lived beyond the age of 95 reached AgeMax about 15 years later (around age 75), and experienced a slower decline in weight, compared to participants with a conventional lifespan. The older AgeMax and flatter Slope were both significant predictors of better survival after age 80 and increased chances of extreme longevity. Our findings suggest that patterns of long-term changes in body weight can be informative biomarkers of aging and longevity. A slower or postponed decline in weight at the oldest old ages may indicate better physical resilience contributing to an organism’s ability to reach longevity limits.

